# Combined transcriptomic and metabolomic analyses of high temperature stress response of quinoa seedlings

**DOI:** 10.1186/s12870-023-04310-y

**Published:** 2023-06-01

**Authors:** Heng Xie, Ping Zhang, Chunhe Jiang, Qianchao Wang, Yirui Guo, Xuesong Zhang, Tingzhi Huang, Junna Liu, Li Li, Hanxue Li, Hongxin Wang, Peng Qin

**Affiliations:** 1 College of Agronomy and Biotechnology, Yunnan Agricultural University, Kunming, 650201 China; 2 Academic Affairs Office, Yunnan Agricultural University, Kunming, 650201 China

**Keywords:** Stress response, Metabolomics, Transcriptomics, Tolerance, Purine metabolism, Quinoa

## Abstract

**Background:**

Quinoa (*Chenopodium quinoa* Willd.) originates in high altitude areas, such as the Andes, and has some inherent characteristics of cold, drought, and salinity tolerance, but is sensitive to high temperature.

**Results:**

To gain insight into the response mechanism of quinoa to high temperature stress, we conducted an extensive targeted metabolomic study of two cultivars, Dianli-3101 and Dianli-3051, along with a combined transcriptome analysis. A total of 794 metabolites and 54,200 genes were detected, in which the genes related to photosynthesis were found down-regulated at high temperatures, and two metabolites, lipids and flavonoids, showed the largest changes in differential accumulation. Further analysis of the Kyoto Encyclopedia of Genes and Genomes (KEGG) pathway and transcription factors revealed that quinoa inhibits photosynthesis at high temperatures, and the possible strategies being used for high temperature stress management are regulation of heat stress transcription factors (*HSFs*) to obtain heat tolerance, and regulation of purine metabolism to enhance stress signals for rapid response to high temperature stress. The tolerant genotype could have an enhanced response through lower purine levels. The induction of the stress response could be mediated by *HSF* transcription factors. The results of this study may provide theoretical references for understanding the response mechanism of quinoa to high temperature stress, and for screening potential high temperature tolerant target genes and high temperature tolerant strains.

**Conclusions:**

These findings reveal the regulation of the transcription factor family *HSF* and the purinergic pathway in response to high temperature stress to improve quinoa varieties with high temperature tolerance.

**Supplementary Information:**

The online version contains supplementary material available at 10.1186/s12870-023-04310-y.

## Background

Quinoa (*Chenopodium quinoa* Willd.) is an annual dicotyledonous plant of the subfamily Quinoa, family Amaranthaceae, cultivated mainly at high altitudes in Argentina, Bolivia, Chile, Colombia, Ecuador and Peru for almost 7,000 years [1, 2]. Quinoa is considered a pseudo-cereal due to its seed characteristics—its seeds have more protein and show a balanced distribution of essential amino acids as compared to most cereals. Quinoa is recognized by the Food and Agriculture Organization as the only monocrop that can meet the nutritional needs of the human body and is admired as one of the food security crops of the century [3]. Quinoa can withstand a vast majority of abiotic stresses, such as high salinity and drought, making it adaptable to most climatic regions [4]. The main breeding objectives for quinoa improvement currently include dwarfing the plant, making more compact strains, and improving the tolerance to heat and biotic stresses [5, 6].

High temperature stress (HS) is a common abiotic stress in plants during growth and development, which causes disturbances in plant metabolism by degrading proteins, thereby resulting in stunted growth and development [7]. HS can be classified into direct injury, which causes rupture of cell structures and tissue necrosis, and indirect injury, which disrupts biofilm systems and affects metabolic rates [8]. Under non-irrigated conditions, heat-only stress facilitates leaf carbon assimilation capacity, but increases plant water demand, which in turn may lead to plant water stress and yield reduction [9]. Tovar (2020) et al., [10] differentially heated either the roots or branches of quinoa and found that yield loss for the branches was attributable to heat stress. They observed that plants with heated branches matured later and had higher biomass of non-reproductive branches as compared to the control, whereas plants with both heated roots and branches had higher yields of spikes that escaped high temperatures [10]. This study suggests that quinoa employs an evasion strategy to resist HS. A better understanding of the mechanism of plant response to HS can help breed new varieties with better performance. Therefore, it is significant to explore the effects of HS on the defense mechanism of the plants.

Plants under HS initiate a series of defense mechanisms in response to these adverse effects, such as regulation of metabolic pathways, modulation of transcription levels, and increased levels of antioxidants, osmoprotectants, and stress protein responses, especially the expression of heat shock proteins (HSPs) [11]. In general, heat stress-induced responses in plants include altering photosynthetic mechanisms, changing cell structure to maintain membrane function, and regulating the expression of transcription factors, phytohormones, heat shock proteins, and metabolic synthetic pathways [12]. Recently, purine metabolism was found to be associated with signal recognition, transcription, stress, and lipid binding [13]; purine nucleotide play an important role in stress response and plant development [14]. The transcription factors associated with HS are *PIF4*, *HSF*, and *WRKY* [15, 16]. Heat stress transcription factors (*HSF*) and heat shock proteins (HSPs) comprise the classical heat stress responses by recognizing conserved heat shock elements (HSEs) in their promoters, which are responsible for activating heat response genes (e.g. Hsp genes). Eukaryotic response to high temperatures involve heat stress transcription factors (*HSFs*) and multiple signaling pathways, which play an important role in the regulating responses to extreme heat stress [17].

The following three questions form the focus of this study on the response mechanism of quinoa under HS: (1) Which metabolites are more affected in HS? (2) Which metabolic pathways respond to HS? and (3) Which HSFs are differentially expressed under high temperature? In this study, two independently selected black quinoa cultivars, Dianli-3101 and Dianli-3051, were used as test materials. We integrated non-targeted metabolomic and transcriptomic approaches with bioinformatic methods to explore the resistance mechanism of quinoa under HS to uncover key metabolic pathways and key regulatory factors, and to provide theoretical references for further selection of better quinoa varieties with stable and high yield under HS.

## Results

### Changes in agronomic traits of quinoa seedlings under high temperature stress

**Fig. 1:**
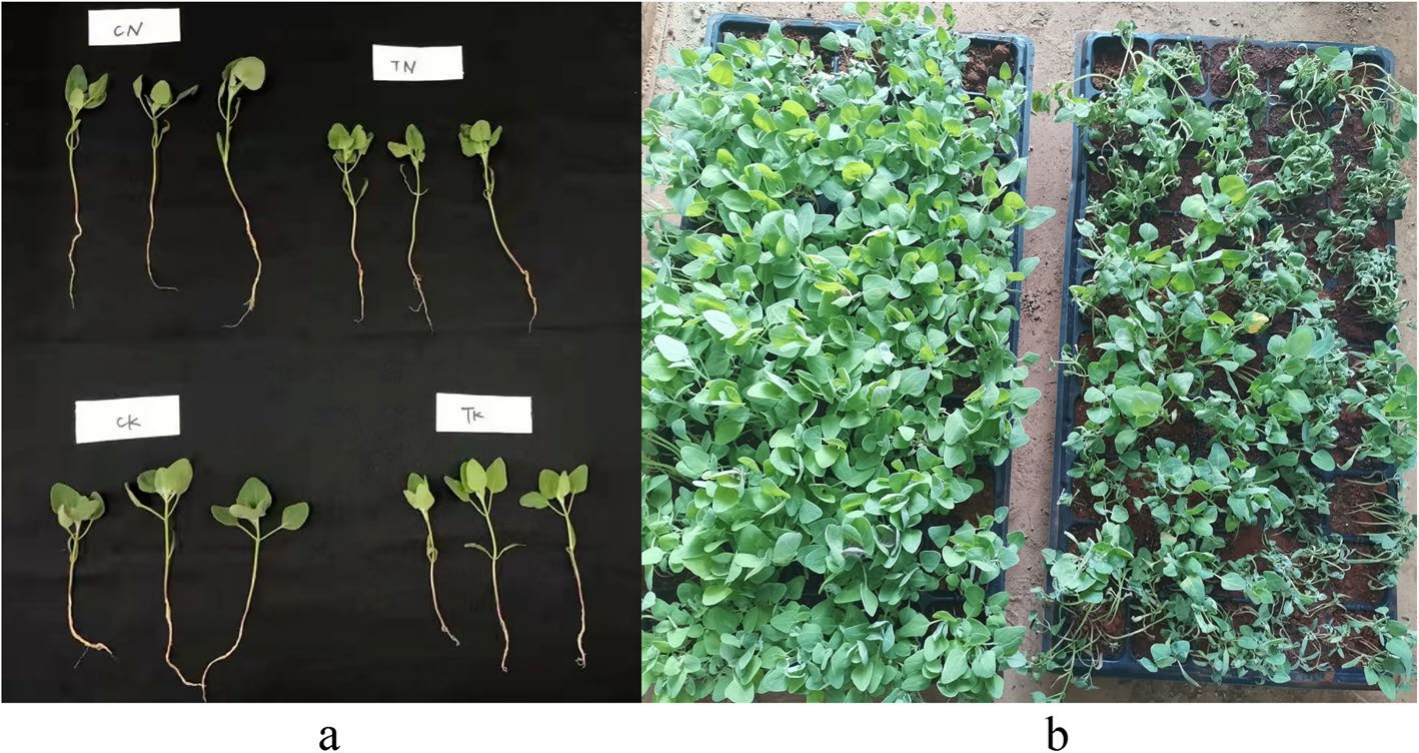
Phenotype of quinoa after 4 days of HS: **a** Comparison of single plants of quinoa under HS and **b** Overall comparison of quinoa under HS

We compared the changes in morphological indicators of Dianli-3101 and Dianli-3051 under HS (Fig. 1). The absolute value of red-green value a was greater, but brightness (L) was smaller in Dianli-3101 than that in Dianli-3051 at high temperature. The root systems of both cultivars were smaller at high temperature than that at 40℃. Presumably, the root growth was inhibited at high temperature. In Dianli-3101, the root length was greater than that of Dianli-3051, whereas the variation in plant height was smaller than that of Dianli-3051. The relative water content and root to crown ratio of Dianli-3101 were more stable and plant height variation at high temperature was smaller than that of Dianli-3051. Thus, Dianli-3101 was observed to be more adaptable at high temperature (Table 1).

**Table 1:** Agronomic traits of quinoa under different treatments

Sample name	Height(cm)	Root length(mm)	Leaf area(mm^2^)	Relative moisture content	Root-to-crown ratio	Leaf color brightness (L)	Leaf color red-green value (A)
	M ± SD	M ± SD	M ± SD				
TK	11.4 ± 1.16^c^	124.64 ± 23.01^b^	284.01 ± 39.03^a^	92.1%	3.31%	37.1	-6.3
CK	13.0 ± 1.38^bc^	234.49 ± 48.66^a^	265.69 ± 44.06^a^	91.9%	4.42%	35.3	-5.6
TN	13.8 ± 0.76^b^	108.30 ± 10.05^b^	157.30 ± 15.40^b^	89.3%	2.74%	38.8	-4.5
CN	16.6 ± 1.01^a^	246.89 ± 27.98^a^	194.56 ± 24.00^b^	91.2%	4.66%	36.6	-4.6

TK, CK, TN, CN specific details are shown in Table 3. Agronomic traits sampled at the six-leaf stage of quinoa, where (L) denotes brightness, (A) denotes red and green values, and the negative numbers denote green values. The lower the green luminance, the higher the luminance. The mean ± standard deviation is M ± SD, different lowercase letters denote a significant difference at the 0.05 level (*P* < 0.05)

### Transcriptome analysis of quinoa seedling

#### Transcriptome sequencing and database analysis

**Fig. 2:**
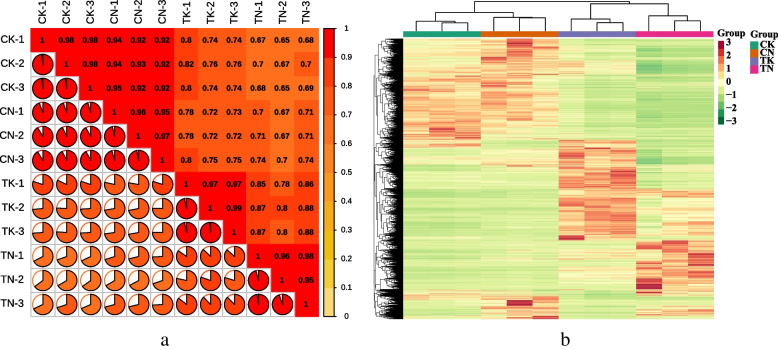
Quantitative analysis of gene expression in quinoa lines under high temperature stress: **a** correlation heat map. **b** Heat map of differential gene clustering

After transcript reconstruction by BLAST software, the unigenes found in the sequencing results that did not belong to the reference genome were defined as novel genes. A total of 6,530 quinoa-specific novel genes were analyzed by novel gene mining. The new genes were compared and functionally annotated with Kyoto Encyclopedia of Genes and Genomes (KEGG, https://www.kegg.jp/kegg/compound/), Gene Ontology (GO), NR, Swiss-Prot, trEMBL and Karyotic Orthologous Groups (KOG) databases using BLAST software (Table S1). In this experiment, the Pearson’s Correlation Coefficient (R2) of biological replicate sequences was greater than 0.9 in different group comparisons (Fig. 2a), and the correlation between replicate samples was strong. We used the fragment per kilobase of transcript per million mapped reads (FPKM) value to express the gene expression level, performed hierarchical cluster analysis, and plotted the clustering heat map for each differential grouping. We observed significant differences in gene expression among the different groups in this experiment (Fig. 2b). These results indicated the high reliability of RNA-Seq data.

### Functional annotation and enrichment analysis of differentially expressed genes

**Fig. 3:**
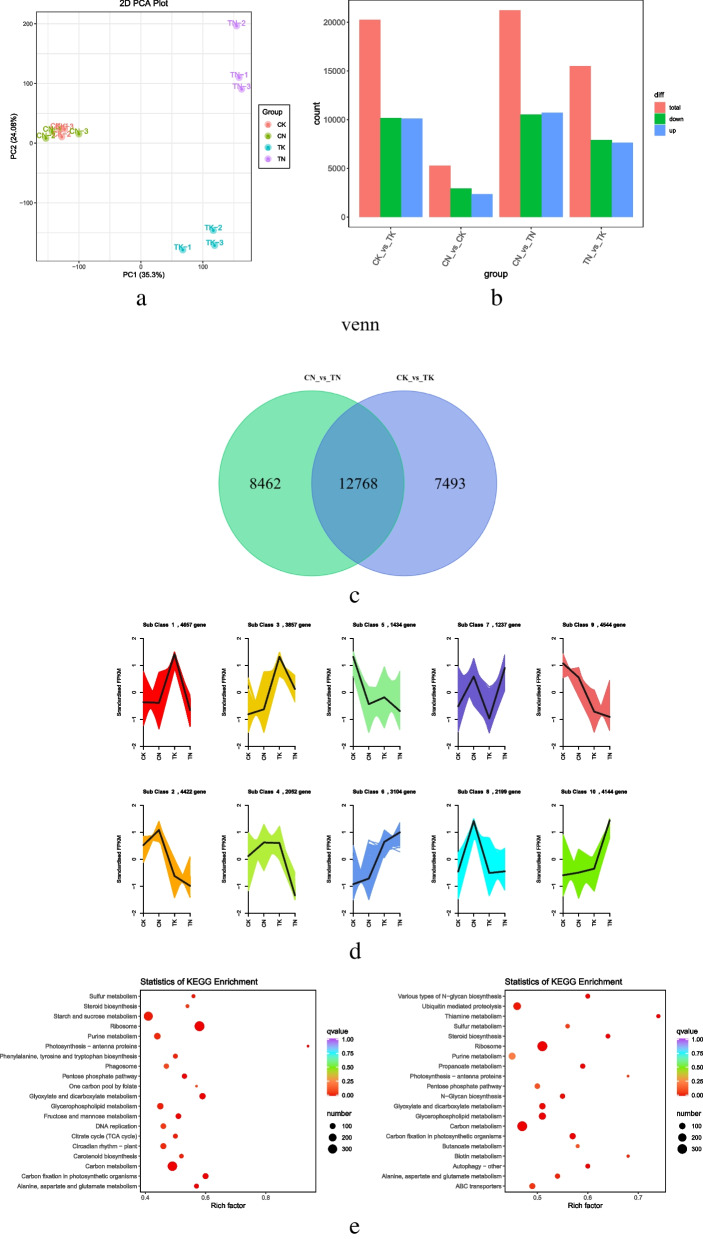
Changes in DEG expression. **a** PCA plot of DEGs: **b** Statistical plot of DEGs: yellow boxes indicate up-regulation, blue boxes indicate down-regulation. **c** Differential gene Wayne maps, non-overlapping areas represent differential genes specific to that differential subgroup, overlapping areas represent differential genes common to several differential subgroups that overlap. **d** K-Means clustering plot, the horizontal coordinate represents the sample, and the vertical coordinate represents the centralized and normalized expression values. **e** KEGG enrichment scatter plot

Differentially expressed genes (DEGs) were identified based on their expression in different samples; these were subjected to comparative analysis and enrichment analysis. We divided the samples into four groups: CK, TK, CN, and TN with three biological replicates in each group. Comprehensive principal component analysis (PCA) (Fig. 3a) showed that the biological replicates within the groups were good, and the samples differed significantly between the high temperature treatment group and normothermia group. The samples in the two groups differed significantly after the high temperature treatment. Comparative analysis was performed according to DESeq2 analysis with |log_2_(fold change)| ≥ 1 and false discovery rate (FDR; error detection rate) < 0.05 as screening conditions. A total of 20,260 DEGs were detected in CK VS TK; 10,104 DEGs were up-regulated and 10,156 DEGs were down-regulated in high temperature treatment compared to room temperature. 21,230 (10,708 up-regulated; 10,522 down-regulated) DEGs were found in CN VS TN. A total of 15,509 DEGs were detected in the sensitive line TN vs. TK (up-regulated 7,612; down-regulated 7,897 DEGs in the high-temperature tolerant types)(Fig. 3b). Venn diagram (Fig. 3c) analysis showed 12,768 DEGs in the two control groups. k-mean cluster analysis was used to classify all the DEGs into 10 subclasses: subclass 1 was the largest, containing 4,657 DEGs, whereas subclass 7 was the smallest, containing 1,237 DEGs (Fig. 3d).

To further analyze the DEGs, we subjected them to GO and KEGG enrichment analysis. GO clustering analysis of DEGs revealed that the DEGs in the three comparison groups were mainly concentrated in three major categories: biological processes (BPs), molecular functions (MFs), and cellular components (CCs) (Supporting Information, Fig. S1). The up-regulated DEGs were mainly involved in the negative regulation of biosynthetic processes and response to heat (BPs), calmodulin binding and signaling receptor activity (MFs), and ubiquitin ligase complexes and endosomal membranes (CCs). The down-regulated DEGs were mainly involved in photosynthesis (BPs), purine nucleoside binding (MFs), and photosystems (CCs). KEGG enrichment analysis showed that in the high temperature treatment group (C vs. T), the top 20 metabolic pathways before enrichment included carbon metabolism; ethoxylate and dicarboxylate metabolism; carbon fixation by photosynthetic organisms; alanine, aspartate and glutamate metabolism; and the pentose phosphate pathway (Fig. 3e). ‘Carbon metabolism’, ‘nicotinate and nicotinamide metabolism’, and ‘propanoate metabolism’ were significantly enriched in TN vs. CK. The biological activity of quinoa seedlings under heat stress may be maintained mainly through the regulation of carbon and organic acid metabolism, whereas the biological activity of heat-tolerant plants may depend mainly on the enhancement of carbon metabolism, propionate metabolism, and nicotinic acid and nicotinamide metabolism to resist heat stress.

### Transcription factors (TFs)

**Fig. 4:**
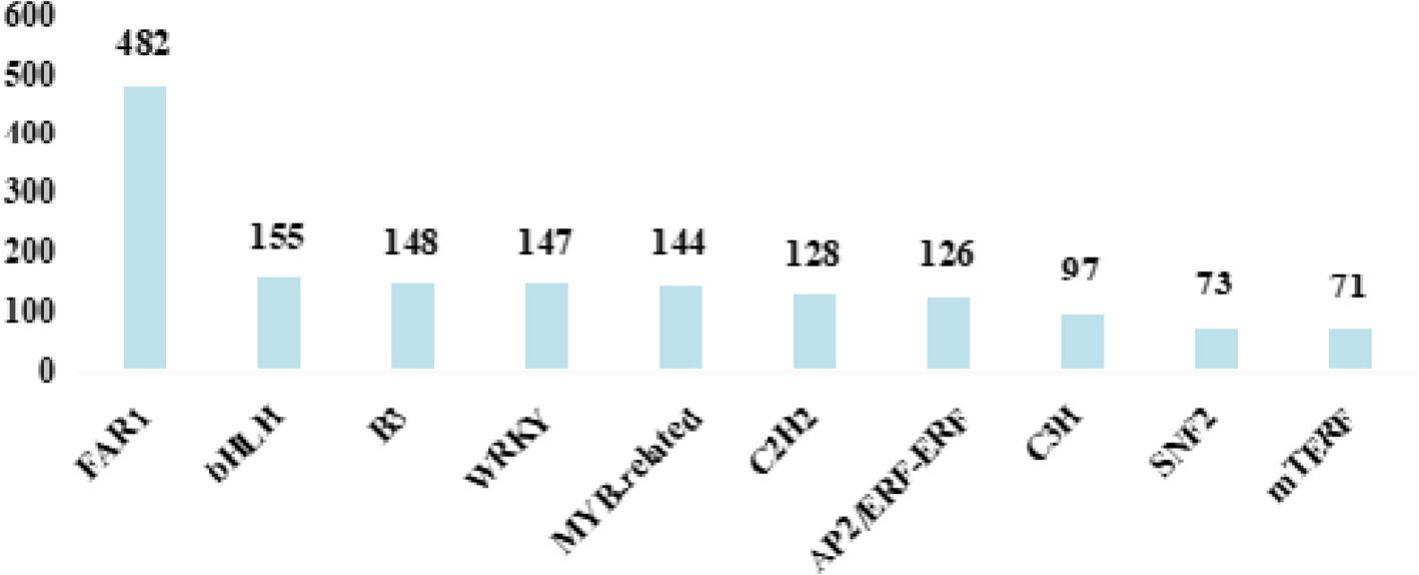
Transcripts of the top ten TF-encoding genes

The transcripts of TF-encoding genes were further investigated to explore the regulatory mechanisms of quinoa subjected to HS. We identified 3,263 TF-encoding genes from 92 different families (Table S2). The top 10 TFs were *FAR1* (482), *bHLH* (155), *B3* (148), *WRKY* (147), *MYB-related* (144), *C2H2* (128), *AP2/ERF-ERF* (126), *C3H* (97), *SNF2* (73), and *mTERF* (71) families (Fig. 4).

### Qualitative and quantitative analysis of relevant metabolites

**Fig. 5:**
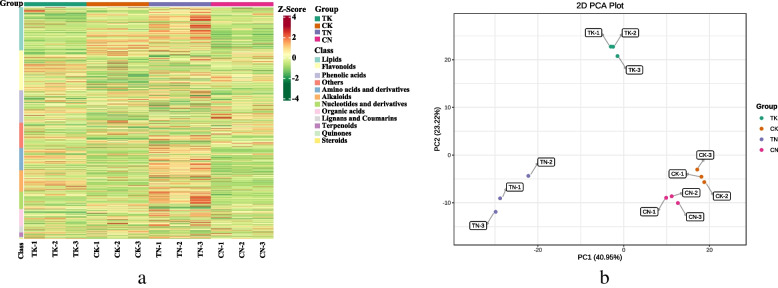
Quantitative analysis of metabolome of quinoa lines under high temperature stress: **a** Overall clustering map of samples. **b** PCA plot of subgroups

Overlap display analysis of the total ion current plots analyzed using mass spectrometry of different QC samples showed a high overlap of the total ion current metabolite detection curves, indicating good technical reproducibility of metabolite extraction and detection (Fig. S2). In this part of the study, the samples were divided into four treatment groups, TK, TN, CK and CN, and the metabolite contents were normalized to construct a hierarchical clustering heat map (Fig. 5a). Subsequently, PCA was performed; and PC1 and PC2 explained more than 64% of the variation, mainly distinguished by PC1 (Fig. 5b). The results showed a significant separation between high-temperature and normothermic treatments. The differences between heat-tolerant and heat-sensitive cultivars were significantly greater at high temperature than those at normothermia. Orthogonal projection-discrimination analysis (OPLS-DA) was performed on the potential structures, and the scoring results showed that the Q2 values of CK vs. TK, CN vs. TN, and TN vs. TK samples were 0.983, 0.979, and 0.982, respectively, indicating that the model was very stable and reliable (Fig. S3).

### Metabolite content analysis

**Fig. 6:**
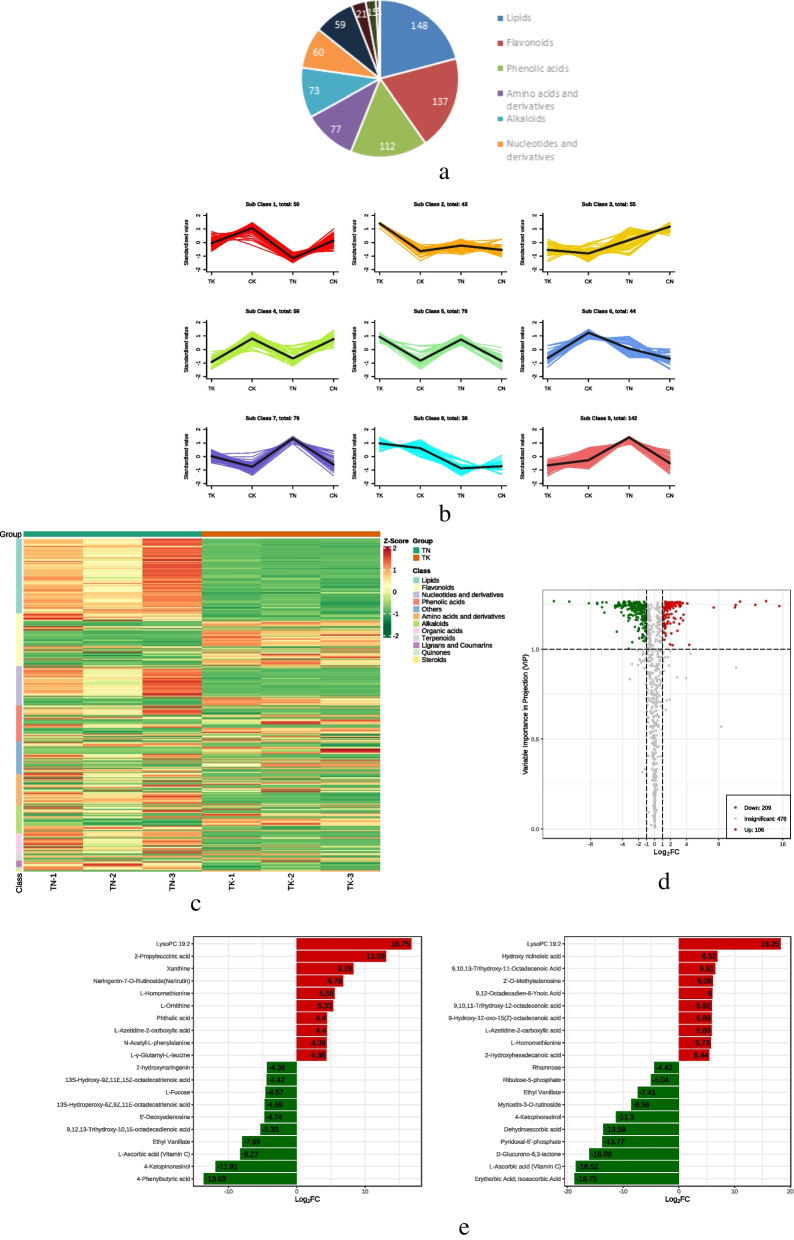
Metabolite content analysis of quinoa lines under high temperature stress: **a** pie chart of various differential metabolite contents. **b** K-Means plot of differential metabolites. **c** heat map of differential metabolites. **d** volcano plot of differential metabolites. **e** histogram of differential multiplicity of DAMs

The ultra-high liquid chromatography and tandem mass spectrometry (UPLC-MS/MS) platform and MetWare database (Wuhan, China) were used to detect the metabolites. We detected 794 metabolites and divided them into 11 types. Lipids were the most diverse with 148 species, followed by flavonoids and phenolic acids with 137 and 112 species, respectively (Fig. 6a).

We screened differential metabolites (DAMs) based on multivariate OPLS-DA models for variable importance projection (VIP ≥ 1) and difference multiplicity (log_2_|Foldchang| ≥ 1). The DAMs were subjected to z-score normalization of the relative content of substances, followed by K-means clustering analysis, and the differential metabolites were classified into 9 clusters using K-Means aggregation analysis (Fig. 6b, Table S3). We observed 315 (209 up-regulated and 106 down-regulated) in TN vs. TK, 301 (160 up-regulated and 141 down-regulated) in CK vs. TK, and 382 DAMs (289 up-regulated, and 93 down-regulated) in CN vs. TN. In TN vs. TK, lipids and flavonoids accounted for 22.5% and 5.8% of the metabolites, respectively (Fig. 6c). The DAMs of both the comparison groups clearly reflected the above changes (Fig. 6d). In the CK vs. TK and CN vs. TN groups, 119 metabolites showed a significant change in abundance. Compared to the heat-tolerant strains, the heat-sensitive strains showed greater changes in metabolite abundance, with lipids showing the greatest rise and flavonoids the most pronounced decline. Based on the changes in fold change of metabolite accumulation, we identified the top 10 DAMs that increased or decreased in the CK vs. TK and CN vs. TN groups (Fig. 6e); and via screening, we found that the co-accumulation of six metabolites changed in all the groups (Lipids, amino acids and their derivatives were the main substances whose accumulation increased, while the accumulation of flavonoids and phenolic acids decreased). The accumulation of lysophosphatidylcholine 19:2, L-homothionine, and L-acridine-2-carboxylic acid increased; that of 4-ketoresinol, L-ascorbic acid(vitamin C), and ethyl vanillate decreased. Notably, most of the substances that accumulated differently in heat-tolerant strains were related to energy metabolism (Fig. 6e).

### KEGG enrichment analysis of DAMs

We enriched the measured DAEs to the KEGG pathway and used *P*-value < 0.05 as the screening condition. We found that purine metabolism, arginine biosynthesis, and ABC transporter protein were significantly enriched in both cultivars under high temperature. Amino acid biosynthesis and valine, leucine and isoleucine degradation were enriched only in CK vs. TK. Arginine biosynthesis and glutathione metabolism were associated with more abundant DAMs in CN vs. TN (Fig. S4).

### Joint analysis of transcriptome and metabolome

#### Changes in gene expression and metabolite accumulation in quinoa under high temperature stress

**Fig. 7:**
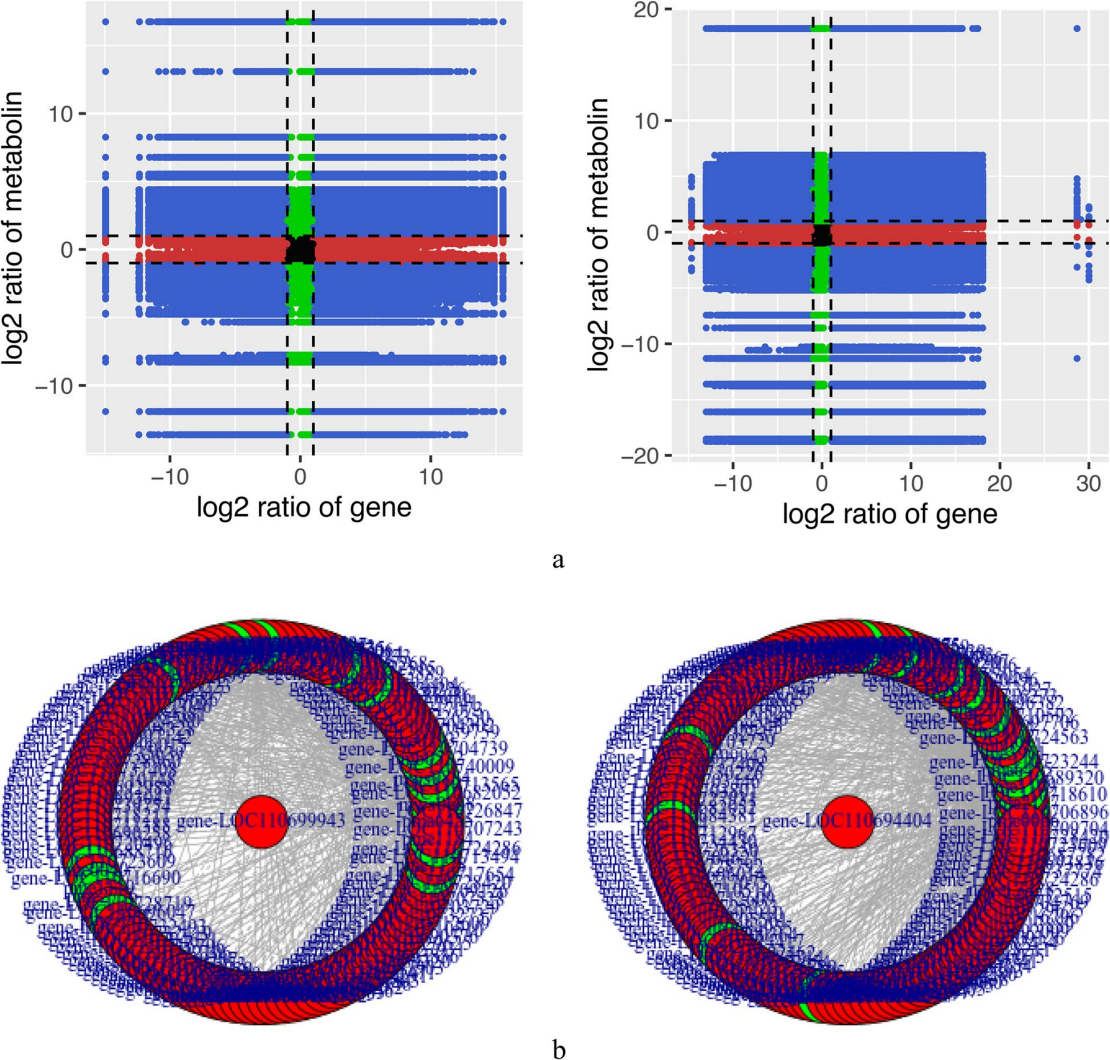
Combined gene and metabolite analysis: **a** Correlation analysis nine quadrant diagram **b** Correlation network diagram

We performed a combined transcriptome and metabolome analysis to further understand the response mechanism of quinoa to HS. Correlation analysis showed that several genes were strongly positively correlated with metabolites (R > 0.8) in the comparison between CK vs. TK and CN vs. TN (Fig. 7a). These results suggest that the changes in metabolite accumulation may be regulated directly or indirectly by the corresponding genes. CK vs. TK and CN vs. TN showed one common significantly enriched pathway (purine metabolism) (Fig. S5). Correlation analysis of DEGs with DAMs in the purine metabolism pathway (Fig. 7b) revealed that the genes LOC-110,699,943 and LOC-110,694,404 were closely associated with several key metabolites in the pathway. LOC-110,699,943 and LOC-110,694,404 co-regulate the down-regulate the expression of the enzyme phosphoglucomutase. These genes also function in pathways related to soluble sugar and amino acid metabolism, with phosphoglucomutase positively correlated with ribulose-5-phosphate levels (*R* = 0.918) and negatively correlated with L-glutamine levels (*R* = -0.953) (Table S4). Furthermore, under HS, the content of soluble sugars, such as glucose and fructose, decreased only in the CN vs. TN group. Correlation analysis showed that specific genes were highly correlated with purine metabolites, suggesting that these purine synthesis genes play a crucial role in purine synthesis under HS. The above results suggest that HS may significantly alter the expression of purine and soluble sugar-related genes and the accumulation of metabolites in quinoa. We hypothesize that in heat-sensitive cultivars, HS may affect the content of soluble sugars by regulating purine metabolism.

### Purine metabolism in response to heat stress in HS

**Fig. 8:**
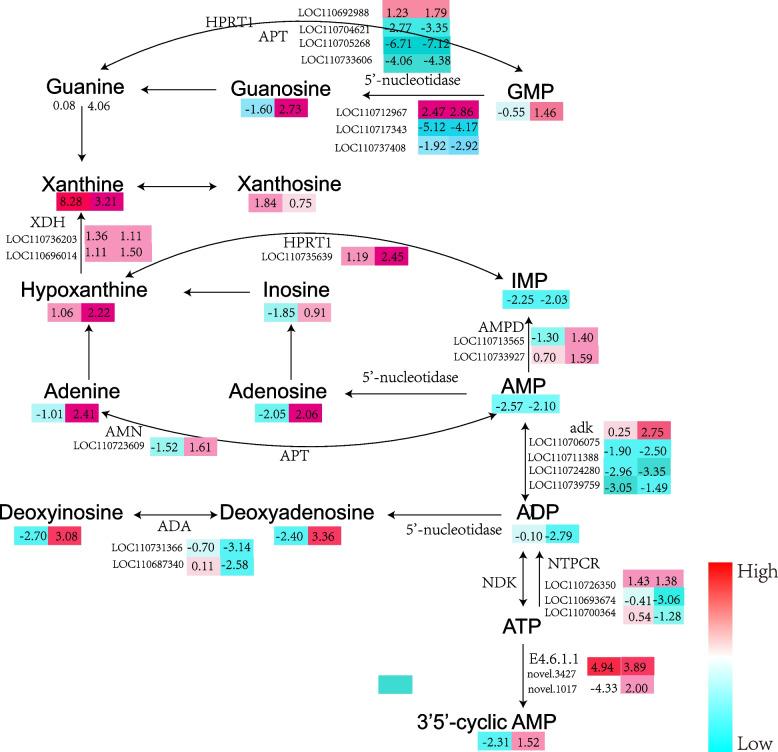
Main pathways of purine metabolism under HS in quinoa: The gene expression level and the metabolite value expressed by the log2FC values of different groups are compared. APT: adenine phosphoribosyltransferase; HPRT1: ypoxanthine phosphoribosyltransferase; XDH: xanthine dehydrogenase; AMN: AMP nucleosidase; AMPD: AMP deaminase; ADK: adenylate kinase; ADA: adenosine deaminase; NDK: nucleoside-diphosphate kinase

In our study, we analyzed the differences between CK and TK, and CN and TN groups and the dynamics of the metabolites. Based on the KEGG database, we performed enrichment analysis of DAMs and DEGs associated with purine metabolism in CKvs.TK and CNvs.TN. Based on previous studies　and combining the KEGG purine metabolism pathway (https://www.kegg.jp/pathway/ko00230), we have, for the first time, proposed the key pathways of purine metabolism biosynthesis in quinoa under HS (Fig. 8). They are essential for maintaining or restoring protein endostasis. Gene-metabolite correlation networks can be used to elucidate functional relationships and identify novel regulatory factors; and Pearson correlation coefficients were determined for the DEGs and DAMs associated with purine metabolism.

Purine metabolism refers to the metabolic pathways that synthesize and catabolize purines present in many organisms [18]. KEGG enrichment analysis showed that the purine metabolism pathway was significantly enriched during HS (Fig. 3e). All differentially accumulated metabolites related to purine metabolism were upregulated. Meanwhile, the enrichment analysis of DAMs and DEGs showed that purine metabolism was co-mapped based on the results of the KEGG database. Under HS, 123 DEGs were highly correlated with 16 metabolites (R2 > 0.8, P < 0.05) (Fig. 7b). Among them, 5′-nucleotidase, adenine phosphate ribosyltransferase (APT), and hypoxanthine ribosyltransferase (HPRT1) were the key enzymes in the enzymatic reactions of purine metabolism biosynthesis. The expressions of 5′-nucleotidase (LOC110717343 and LOC110737408) and APT (LOC110704621, LOC110705268, and LOC110733606) were down-regulated, whereas the enzyme HPRT1 (LOC110735639) was up-regulated in both the cultivars under HS. 5′-nucleotidase regulates the endogenous cycle of substances by regulating three metabolic nodes that catalyze the formation of deoxyribonucleotides from monophosphate; and APT and HPRT1 recycle adenine, guanine, and hypoxanthine to AMP, GMP, and IMP, respectively. HS causes a significant downregulation of the expression of most genes in the pathway, especially in heat-sensitive strains. Deoxyinosine, deoxyadenosine, guanosine, adenosine, and 3′,5′-adenine nucleotide (3′,5′-Cyclic AMP) levels decreased in the heat-tolerant strain and increased in the heat-sensitive strain. Although the pathways that regulate purine metabolism biosynthesis have been identified, the genes playing the key role in regulating the purine metabolism biosynthesis pathway are still unclear.

### qRT-PCR analysis of gene expression

To further validate the RNA-Seq results, we randomly selected nine genes involved in the purine metabolism pathway from the candidate DEGs and analyzed them using RT-qPCR to validate the RNA sequencing transcriptome data. The RT-qPCR results of the candidate genes were generally consistent with the relative transcript abundance found in the transcriptome analysis (Fig. S6), indicating the reliability of the RNA-Seq data. Detailed primer information is presented in Supporting Information, Table S5.

### Expression of*HSF* in quinoa under high temperature and its relationship with heat response

**Fig. 9:**
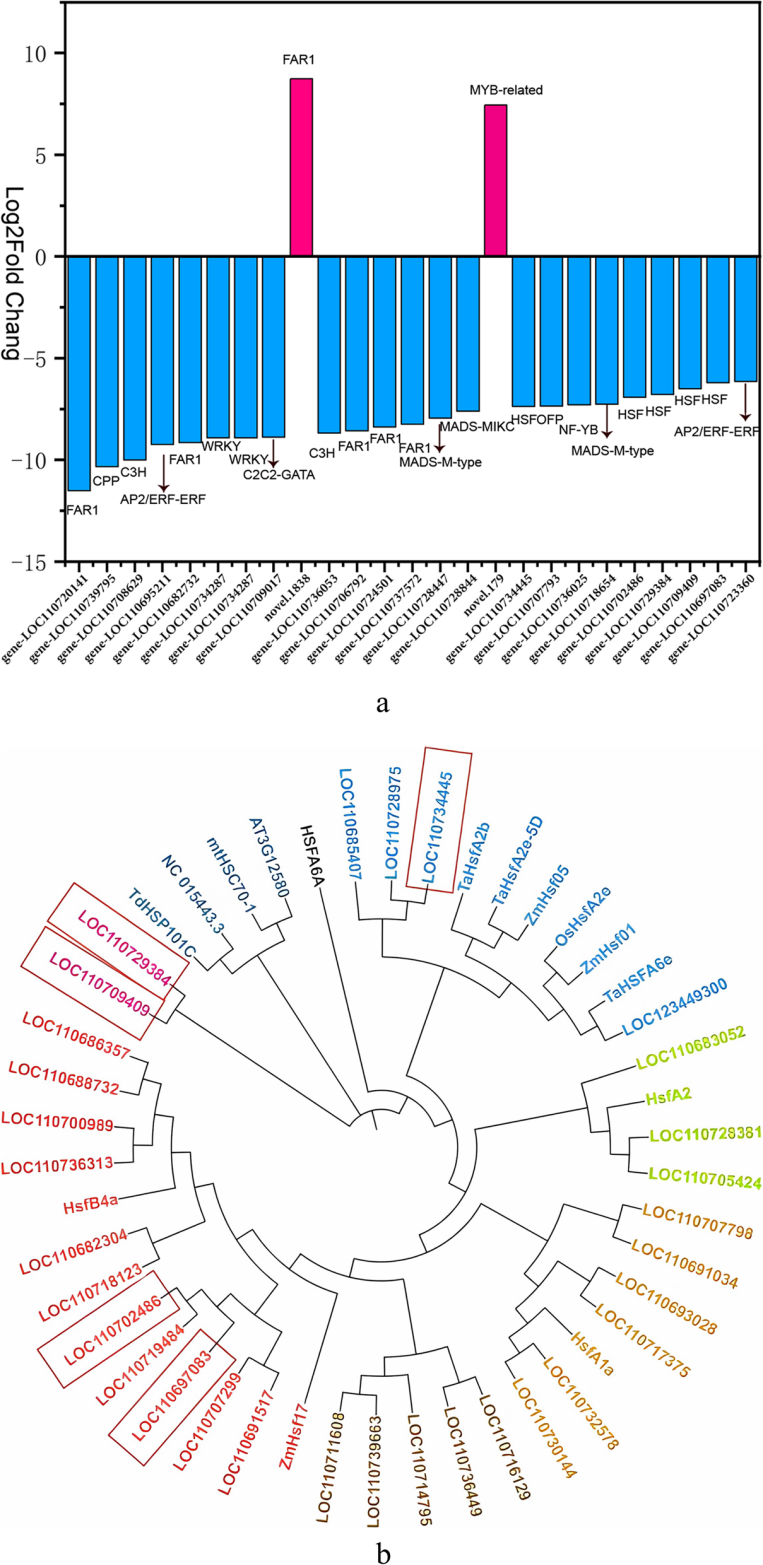
Differential expression of quinoa-encoded genes under HS: **a** The top 25 TF-encoded genes with the largest fold changes in TNvsTK. **b** Phylogenetic tree of *HSF* transcription factors from different sources: *HSFA6A, AT3G12580, mtHSC70-1*(Arabidopsis), *ZmHsf05, ZmHsf01, ZmHsf17* (*Zea mays*),*NC_015443.3,HsfA1a,HsfA2* (tomato), *HsfB4a,TdHSP101C,HSFA6A,TaHsfA2e-5D,TaHsfA2b* (bread wheat)

Approximately 882 differentially expressed TF-encoding genes were identified in TN vs. TK, with the ratio of up-regulated to down-regulated genes being 268:614, respectively. These transcription factors were mainly from different gene families such as *AP2/ERF, MYB-related, WRKY, FAR1, C2H2, bHLH, C3H, bZIP, MYB*, and *HSF*. The *AP2/ ERF, MYB-related*, and *WRKY* families were the most abundant TFs. The top 25 TF-encoding genes with the largest fold changes in TN vs. TK are shown in Fig. 9a. Five of the top 25 differentially expressed TF genes (LOC110734445, LOC110729384, LOC110709409, LOC110702486, and LOC110697083) belonged to the *HSF* family. Although the abundance of *HSF* was not as high as the other families, it was highly variable. This suggests that TFs of *HSF* are actively expressed in quinoa under HS.

We used transcriptome sequence comparison and identified 31 Hsf-encoding genes in the *HSF* family. To analyze the response of quinoa *HSFs* to HS, 20 differentially expressed *HSFs* in the TN vs. TK group were selected for further analysis in this study, using *HSFs* downloaded from the NCBI database (Table S6). Although plant *HSFs* have a highly conserved structure, their remarkable diversity among plants reflects their numerous functions [19]. We selected Arabidopsis, maize, barley, tomato and wheat species for *HSF* analysis (Table 2). Phylogenetic analysis revealed the phylogenetic relationships of these *HSF* genes, along with identifying *HSFs* in other crops (Fig. 9b), providing a basis for functional analysis and molecular breeding of quinoa *HSF* genes.

**Table 2:** Overview of plant-related *HSF*s or proteins in response to high temperature stress

Gene/Protein	Source of gene	Stress responses	References
*HSFA6A*	Arabidopsis	Can provide abiotic stress tolerance by regulating ROS homeostasis in plants.	Wenjing W. et al. 2020 [20]
*HSFA1s*	Arabidopsis	The *HSFA1* gene is a major regulator of HSR and an important component of other abiotic stress responses.	Liu HC. et al. 2011 [21]
*mtHSC70-1*	Arabidopsis	Has a role in the establishment of cytochrome c oxidase (COX)-dependent respiration and redox homeostasis in Arabidopsis thaliana	Zhai XT. et al. 2020. [22]
*ZmHsf05*	Zea mays	*ZmHsf05* plays an important role in both basal and acquired temperature tolerance in plants.	Li GL.et al. 2019 [23]
*ZmHsf01*	Zea mays	Lines overexpressing *ZmHsf01* showed higher chlorophyll content and survival rate after HS	Zhang H. et al. 2020 [24]
*ZmHsf17*	Zea mays	Binds to the promoter of small heat shock proteins	Qi H. et al. 2022 [25].
*HSFA1a*	tomato	HSFA1-type *HSF* can promote its activity in HSE for stress protection	Mesihovic A, et al. 2021. [26]
*HsfA2*	tomato	Accumulation of *HsfA2* by moderate HS treatment enhances the ability of seedlings to cope with subsequent severe HS	Fragkostefanakis S. et al. 2016 [27]
*HSFB4a*	bread wheat	Mitigation of *HSFB4a* regulates downstream heat stress response genes to control heat tolerance in heat tolerant species	Rao S.et al. 2022 [28]
*TdHSP101C*	bread wheat	Expression of *TdHSP101* gene is associated with increased heat tolerance	Bento M.et al. 2017. [29]
*TaHSFA6e*	bread wheat	*TaHSFA6e* TF can be used as a promising candidate gene for manipulating heat stress tolerance networks	Kumar RR. et al. 2018. [30]
*TaHsfA2e-5D*	bread wheat	*TaHsfA2e-5D* acts as a positive regulator of plant responses to heat and drought stress	Bi H. et al. 2022. [31]
*TaHsfA2b*	bread wheat	Provides functional heat shock elements which interact with other small molecules	Xue GP. et al. 2014 [32]

## Discussion

HS is one of the key factors affecting plant growth and development and is likely to become increasingly common with global warming [33]. Quinoa is an ancient Andean origin crop, and its native the high altitude barren environment has led to the evolution of tolerance to most abiotic stresses [34], but it is still sensitive to HS. Metabolic pathways in several plants in response to HS have been identified [35, 36] and altered gene expression plays an important role in plant tolerance to HS.

To understand the molecular mechanisms regulating HS tolerance in quinoa, we studied two cultivars, Dianli-3101 and Dianli-3051. We found that plant height, leaf area, and root-to-crown ratio tended to decrease under high-temperature stress, but Dianli-3101 maintained its relative water content more stable under high temperatures (Table 3). We found that the “amino acid biosynthesis” and “degradation of valine, leucine, and isoleucine” pathways were up-regulated in Dianli-3101, and the respiratory pathway, which is normally used as an alternative substrate by plants, was activated, and amino acid biosynthesis promotion enhanced plant stress tolerance. The biosynthesis of amino acids enhances plant stress tolerance [37]. The metabolite abundance of Dianli-3051 was also highly variable, with the greatest rise in lipids and most pronounced decline in flavonoids, which generally have an important role in plant stress resistance [38]. More free fatty acids were added to Dianli-3051 under HS, with the most pronounced increase in lysophosphatidylcholine 19:2 (Fig. 6e), which is an important lipid composition component and plays an important role in the composition of cell membranes [39]. The large increase in lysophosphatidylcholine, indicating the free release of fatty acids from the cell membrane, suggests that the cell membrane conformation of Dianli-3051 may be severely damaged. The initiation of HS affected the expression of numerous genes in the KEGG pathway but mainly affected the expression of metabolic pathways and pathways related to secondary metabolite synthesis (Figs. 3e and S5). These results suggest that Dianli-3101 has better stability at high temperatures. This is because it exhibits greater adaptability at all three levels: morphological, metabolic, and transcriptional. In addition, the transcriptomic analysis revealed a down-regulation in the expression of DEGs involved in photosynthesis (BPs), purine nucleoside binding (MFs), and photosystems (CCs) under HS (Fig. S1). Since the photosynthetic pathway is the main metabolic pathway for energy acquisition in plants, these results suggest that high temperature may limit the growth and development of quinoa by inhibiting photosynthesis.

Through a combined analysis of quinoa genes and metabolites under HS (CK vs. TK, CN vs. TN), we found that purine metabolism had an important role in response to HS (Fig. 7b). In purine metabolism, adenine, guanine, and hypoxanthine were recycled as Adenosine 5’-monophosphate (AMP), Guanine nucleotides (GMP), and Inosine monophosphate (IMP), respectively; and two enzymes, APT and HPRT1, were involved in these remedial pathways (Fig. 8). Among these, APT plays its traditionally described role as a salvage pathway enzyme to restore adenine to AMP, and HPRT1 restores hypoxanthine to IMP. APT activity in recycling nucleoside in the salvage pathway also enables it to regulate the flux of the nucleotide degradation pathway [40]. AMP plays an important role in mediating stress by altering plant hormone homeostasis and energy metabolites [41]. Additionally, alterations in adenine at the cellular level can trigger tolerance and promote growth [42], and purine recycling pathways can play the role of cellular signaling molecules in regulating plant responses to abiotic stresses [43]. In quinoa, the accumulation of AMP, GMP, and IMP was reduced in both the cultivars under high temperature and the expression of the genes LOC110717343, LOC110737408, LOC110735639, LOC110704621, LOC110705268 and LOC110733606, the enzymes associated with these genes, was down-regulated in the enzymatic responses. The purine metabolites were more significantly down-regulated in the heat-tolerant cultivars (Fig. 8). It is possible that triggering adenine supplementation improves tolerance to several stresses and endogenous adenine levels are altered in response to stress [44]. Overall, purines may play a role in regulating signals in response to abiotic stresses and plant growth and may enhance stress signals to rapidly initiate response mechanisms following heat stress.

In the face of heat stress, plants use heat shock response, an ancient signaling pathway, to enhance heat tolerance [45]. *HSFs* are plant-specific genes that activate the expression of heat-stimulated proteins and play an active role in triggering the heat response and mediating heat tolerance [46]. Analysis of TFs in the TN vs. TK group revealed that among the 25 most variable transcription factors, LOC110702486, LOC110697083, LOC110729384, LOC110709409, and LOC11073445 were more significantly differentially expressed in quinoa under heat stress as compared to that under normothermia (Fig. 9a). We hypothesize that these five *HSFs* are the key *HSFs* for triggering heat response and acquiring heat tolerance. By analyzing the phylogenetic relationships of *HSFs* in TN vs. TK with *HSFs* identified in other crops (Fig. 9b), we found that LOC110702486 and LOC110697083 were closely homologous to the wheat heat stress transcription factor *HsfB4a*, LOC110729384 and LOC110709409 were highly homologous to Arabidopsis *HsfA6a* gene, and LOC11073445 is closely related to the wheat heat stress protein *TaHsfA2e-5D*. These results provide a good basis for the cloning of quinoa. Numerous studies report HSF response to high-temperature stress. Wenjing et al. (2020 [20]) found that *HSFA6A* and *HSFA6b* may achieve abiotic stress by regulating plant ROS endostasis tolerance [20]. Bi et al. [31] suggested that *TaHsfA2e-5D* plays a positive regulatory role in plant response to heat and drought stress. Rao et al. [28] found that *HsfB4a* can mitigate the regulation of downstream heat stress response genes to regulate heat tolerance in heat-tolerant species (Table 2). This study suggests that HSF genes are involved in heat stress and reveals the phylogenetic relationship of HSF genes with identified HSFs in other crops. We speculate that LOC110702486, LOC110697083, LOC110729384, LOC110709409, LOC11073445, and other *HSFs* are the core regulators of transcription during HS and are key *HSFs* that enable quinoa to acquire heat tolerance. These results laid the foundation for further understanding of the cloning of these five HSF genes and their functions.

**Fig. 10:**
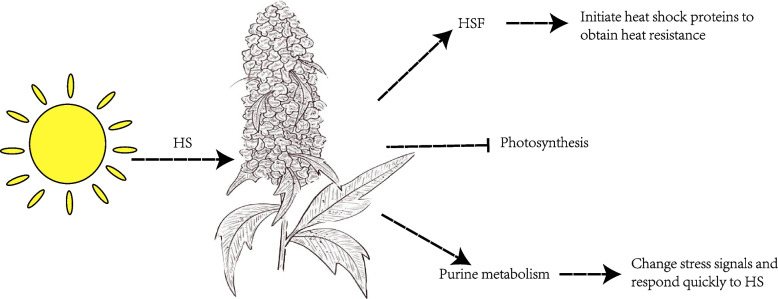
Proposed mechanism of response to high temperature stress in quinoa based on metabolomic and transcriptomic data

This study reveals a possible response mechanism in quinoa under HS (Fig. 10). Quinoa modulates *HSFs* to initiate the expression of heat shock proteins, including initiation of *HSF* genes such as LOC110702486, LOC110697083, LOC110729384, LOC110709409, LOC11073445, and other *HSFs* to obtain tolerance. HS modulates purine metabolic pathways to alter endogenous purine levels that stimulate stress signals and enhance tolerance to heat stress. Heat-tolerant cultivars may trigger enhanced stress responses through lower purine levels, as purine supplementation may enhance stress resistance. However, further studies are needed to fully determine the role of purines in activating plant defenses against abiotic stresses. In the meantime, we suggest that there is a close association between the heat shock response and purine metabolic pathways and that the expression of heat shock proteins may be influenced by purine levels. The results of this study are expected to provide new ideas regarding the molecular response of quinoa under high-temperature stress and molecular breeding.

## Conclusions

we selected two quinoa genotypes, one tolerant, and one sensitive to heat stress, and subjected plantlets to control (22ºC) and heat stress (40ºC) conditions at early development. They observed several physiological traits and took leaf samples to apply metabolomic and transcrptomic combined aprroaches. we found that the purine metabolism pathway was enriched under heat in both genotypes and in both metabolites and gene expression, with lower purine levels under heat stress. we also found that some genes from the transcription factor family *HSF* were overrepresented in the tolerent genotype, and the genes found were homologous to genes from other crops that provide tolerance to abiotic stress. we hypothesize that the heat stress alters the regulation of the purine pathway, resulting in lower purine levels which in turn can triger stress signals. The tolerant genotype could have an enhanced response through lower purine levels. The induction of the stress response could be mediated by *HSF* transcription factors.

## Materials and methods

### Experimental materials

Thirty high-generation quinoa cultivars, selected independently by Yunnan Agricultural University, were used as primary screening material. These cultivars were planted at the Modern Agricultural Education and Research Base of Yunnan Agricultural University in Xundian County, Kunming (102°41′ E, 25°20′ N). Uniform seeds were selected and sown in 50-hole seedling trays (54 cm × 28 cm × 12 cm) with three replicates of each. The first stage was managed according to conventional cultivation management techniques (average temperature: 22 °C; sunshine duration: approximately 10 h; sowing depth: 2–3 cm; loamy soil:humus:organic fertilizer = 1:1:1). When the seedlings reached six-leaf stage, the treatment group was transferred to a greenhouse at 40 °C, the other conditions were maintained the same as those for field planting. The seedlings were subjected to continuous day and night heat treatment of 40 °C. The leaves showed large water-stained scald spots and severe wilting on the fourth day, which was considered the best sampling points. Based on the plant growth and the degree of leaf scald, the heat-resistant (Dianli-3101) and the heat-sensitive (Dianli-3051) strains were selected as samples. The above-ground parts and roots of seedlings were sampled separately; the above-ground parts of seedlings were immediately frozen in liquid nitrogen and stored at − 80 °C. Details of the samples are presented in Table 3. Leaf samples were used for metabolome and transcriptome sequencing and quantitative reverse transcription polymerase chain reaction (RT-qPCR) analysis. Three biological replicates and three technical replicates were included in this study.

**Table 3:** The upper part of the quinoa field is numbered accordingly

Material name	Material characteristics	Processing method	Number Name
Dianli-3101	Heat-resistant type	40℃(Heat damage)	TK
		22℃(room temperature)	CK
Dianli-3051	Thermal type	40℃(Heat damage)	TN
		22℃(room temperature)	CN

### Morphological data acquisition

The treated Dianli-3101 and Dianli-3051 cultivars were sampled separately (three replicates each) to determine plant height, leaf area, dry and fresh weight, root to crown ratio, root length, and leaf color of quinoa seedlings;the height of the plant (distance from the base to the tip of the topmost expanded leaf) of the quinoa seedlings was measured with a Vernier caliper; leaf area was measured using the morphometer of TPYX-A crop leaves (Zhejiang, China, https://www.tpyn.net); root system measurements were made using the GXY-A root analysis system (Zhejiang, China, https://www.tpyn.net); and leaf color was digitized using a color reader (CR-20). Plants were killed at 110 °C for 30 min and then dried at 85 °C to a constant weight for determination of fresh and dry weight and root-to-crown ratio.

### Extensive targeted metabolome analysis

The samples were freeze-dried in a vacuum freeze-dryer and subsequently pulverized in a mixing mill with zirconia beads at 30 Hz for 1.5 min. The lyophilized powder (100 mg) was dissolved in 1.2 mL of 70% methanol solution and vortexed for 30 s, 6 times, every 30 min, and the samples were placed in a refrigerator overnight at 4 °C. After centrifugation at 12,000 rpm for 10 min, the extracts were filtered (SCAA-104, pore size 0.22 μm; ANPEL, Shanghai, China) to obtain the samples for UPLC-MS/MS analysis. Chromatographic separation was performed using an Agilent SB-C18 column (1.8 μm, 2.1 mm × 100 mm) at 40 °C with the mobile phase consisting of pure water with 0.1% formic acid and acetonitrile [47].

The mass spectrometry conditions mainly included: electrospray ionization source temperature of 550 ℃, mass spectrometry voltage of 5500 V, curtain gas of 30 psi, and collision-induced ionization parameters set to high. The metabolite identification annotations were based on the MWDB (Wuhan Metaville Biotechnology Co., Ltd., Wuhan, China, http://www.metware.cn/) database, and the substance characterization was performed based on the secondary spectrum information. The analysis was performed by removing isotopic signals; duplicate signals containing K^+^, Na^+^, and NH_4_^+^ ions. Triple quadrupole mass spectrometry in multiple reaction monitoring (MRM) mode was used for metabolite quantification, and the mass spectral peaks detected for each metabolite in different samples were corrected to ensure accurate qualitative quantification. Quality control samples (QC) are used during instrumental analysis to determine the technical reproducibility of metabolite extraction and detection. PCA and OPLS-DA were used for all identified metabolites to analyze the overall metabolic variation, the variability between groups, and between samples within groups [48]. Based on the results of OPLS-DA, those with VIP ≥ 1 and fold change ≥ 2 or ≤ 0.5 were selected as differential metabolites. The differential metabolites were further calibrated and annotated in the KEGG database. P values from hypergeometric tests were used to determine their significance.

### Transcriptome sequencing and data analysis

Transcriptome sequencing, including RNA extraction, detection, library construction and sequencing, was performed by Wuhan Metware Biotechnology, Ltd. (Wuhan, China, www.metware.cn). RNA integrity and DNA contamination were analyzed using agarose gel electrophoresis. RNA concentration was measured with high accuracy using Qubit 2.0 fluorometer. RNA integrity was detected using Agilent 2100 Bioanalyzer. The obtained mRNA was synthesized into a double-stranded complementary DNA (cDNA), and the purified cDNA was subjected to end repair, A-tailing and sequencing junction ligation, fragment size selection, and finally PCR enrichment to obtain the final cDNA library. Post construction, the library quality was checked and the initial quantification was performed using Qubit 2.0. After the insert size of the library was tested by Agilent 2100, the effective concentration of the library was accurately quantified (effective library concentration > 2 nM) and the library check was completed. Subsequently, sequencing was performed using the Illumina HiSeq platform. The downstream data were filtered to obtain Clean Data, and the Mapped Data was obtained via sequence alignment with the reference genome (v1,WG_genome.fa.gzat https://www.ncbi.nlm.nih.gov/genome/?term%20=Chenopodium+quinoa+willd) [49]. FPKM was used as a measure of transcript or gene expression level [50]. This experiment had biological replicates and differential expression analysis between sample groups was performed using DESeq2. A |log_2_Fold Change| ≥ 1 and FDR < 0.05 were selected as screens for differential genes. After screening for differential genes, expression levels of genes in different sample groups were analyzed for differential expression analysis, functional annotation of differentially expressed genes, and functional enrichment. Functional annotation of differentially expressed genes was performed using KEGG [51], GO, KOG, PfAM, Swiss-Prot, TrEMBL, and NR databases.

### Combined transcriptomics and metabolomics analyses

The same treatment DAMs combined with DEGs were simultaneously mapped onto the KEGG pathway map by analyzing the metabolome and transcriptome results. Correlation analysis was performed for genes and metabolites detected in each sample group, and Pearson correlation coefficients between genes and metabolites were calculated using the COR program in R (Version v1.9.12.31, https://igraph.org/). Network plots were used to represent the correlation coefficients between metabolites and genes, with Pearson correlation coefficients of > 0.8 for each group. For this study, O2PLS models were developed using all the DEGs and DAMs, and based the loading maps selected with different variables with higher correlations; and weights in the dataset were used to filter out the important variables affecting the other group [52].

### RT-qPCR analysis

We used Beacon Designer 7.9 to design 9 primer pairs specific for the selected genes. qRT-PCR was performed using an ABI 7500 system (Applied Biosystems, Foster, CA, USA) and a PerfectStart™ SYBR qPCR Supermix (TransGen Biotech, Beijing, China). Thermal cycling was carried out as follows: 94 °C for 30 s, 40 cycles of 94 °C for 5 s and 60 °C for 30 s. Relative gene expression levels were calculated by the cycling threshold (Ct) 2^–ΔΔCt^ method using TUB-6 as the internal reference gene. Three biological and technical replicates were performed for each group.

### Statistical and sequence analysis

The protein sequences of *HSFs* were downloaded from the National Center for Biotechnology Information (NCBI) database. A bootstrap neighbor-joining evolutionary tree was constructed for phylogenetic analysis using MEGA-X software, and the bootstrap was repeated 1000 times [53]. DNAMAN software (version 9) was used for amino acid sequence comparison.

## Supplementary Information

**Additional file 1.**

## Data Availability

The datasets generated and/or analyzed during the current study are available in the [NCBI] (National Center for Biotechnology Information) repository, [PRJNA939366].We hereby declare that the materials used in this study (Dianli-3101, Dianli-3051) were independently selected and bred by Qin Peng’s group at Yunnan Agricultural University and have the right to use them.

## Abbreviations

HS: High temperature stress
HSPs: Heat shock proteins
HSEs: Heat shock elements
HSFs: Heat stress transcription factors
DEGs: Differentially expressed genes
DAMs: Differential metabolites
PCA: Principal component analysis
TFs: Transcription factors
BPs: Biological processes
MFs: Molecular functions
CCs: Cellular components
OPLS-DA: Orthogonal projection-discrimination analysis
UPLC-MS/MS: Ultra-high liquid chromatography and tandem mass spectrometry
KEGG: Kyoto Encyclopedia of Genes and Genomes
GO: Gene Ontology
