# Supplementary material for: Combined transcriptomic and metabolomic analyses of high temperature stress response of quinoa seedlings
Source: BMC Plant Biol. 2023 Jun 1;23:292. doi: 10.1186/s12870-023-04310-y (PMC10234003; doi:10.1186/s12870-023-04310-y)
Supplement: Supplementary file 1 — Additional file 1. [file 12870_2023_4310_MOESM1_ESM.zip › Support Materials and manuscript/Fig. S1.doc]

CK VS TK

CN VS TN

TN VS TK

**Supporting Information,Fig.S1:**

Classification chart of secondary entries of differential genes.
