# Supplementary material for: Combined transcriptomic and metabolomic analyses of high temperature stress response of quinoa seedlings
Source: BMC Plant Biol. 2023 Jun 1;23:292. doi: 10.1186/s12870-023-04310-y (PMC10234003; doi:10.1186/s12870-023-04310-y)
Supplement: Supplementary file 1 — Additional file 1. [file 12870_2023_4310_MOESM1_ESM.zip › Support Materials and manuscript/Fig.S5.doc]

CKvs.TK

CNvs.TN

**Supporting Information, Figure S5**

KEGG enrichment analysis Histogram：The horizontal coordinate of the bar graph represents the metabolic pathway, the red color in the vertical coordinate represents the enrichment pvalue value of the differential gene, and the green color represents the enrichment pvalue value of the differential metabolite, which is represented by -log(P-value), the higher the vertical coordinate, the stronger the enrichment degree
