# Supplementary material for: Combined transcriptomic and metabolomic analyses of high temperature stress response of quinoa seedlings
Source: BMC Plant Biol. 2023 Jun 1;23:292. doi: 10.1186/s12870-023-04310-y (PMC10234003; doi:10.1186/s12870-023-04310-y)
Supplement: Supplementary file 1 — Additional file 1. [file 12870_2023_4310_MOESM1_ESM.zip › Support Materials and manuscript/Fig.S6.doc]

**Supporting Information**,**Fig. S6. Validation of the transcriptome data using RT-qPCR.**

Line graphs indicate the transcript abundances measured by transcriptome sequencing based on FPKM values. Bar graphs represent the relative expression measured by RT-qPCR expressed in 2^–ΔΔCt^. Data are averages of three replicates and three biological replicates. Error bars indicate SEs.
